# Integrated single-cell transcriptomics of cerebrospinal fluid cells in treatment-naïve multiple sclerosis

**DOI:** 10.1186/s12974-022-02667-9

**Published:** 2022-12-19

**Authors:** Frederike Straeten, Jing Zhu, Anna-Lena Börsch, Baohong Zhang, Kejie Li, I-Na Lu, Catharina Gross, Michael Heming, Xiaolin Li, Rebekah Rubin, Zhengyu Ouyang, Heinz Wiendl, Michael Mingueneau, Gerd Meyer zu Hörste

**Affiliations:** 1 Department of Neurology with Institute of Translational Neurology, Medical Faculty, University Hospital Münster, Münster, Germany; 2 Department of Research, Biogen, Cambridge, MA USA; 3 BioinfoRx., San Diego, CA USA

**Keywords:** Immunology, Multiple sclerosis, Human, Single-cell RNA-sequencing, Central nervous system, Cerebrospinal fluid, Interactive visualization

## Abstract

Multiple sclerosis (MS) is a chronic and often disabling autoimmune disease of the central nervous system (CNS). Cerebrospinal fluid (CSF) surrounds and protects the CNS. Analysis of CSF can aid the diagnosis of CNS diseases, help to identify the prognosis, and underlying mechanisms of diseases. Several recent studies have leveraged single-cell RNA-sequencing (scRNA-seq) to identify MS-associated changes in CSF cells that are considerably more altered than blood cells in MS. However, not all alterations were replicated across all studies. We therefore integrated multiple available scRNA-seq datasets of CSF cells from MS patients with early relapsing–remitting (RRMS) disease. We provide a searchable and interactive resource of this integrated analysis (https://CSFinMS.bxgenomics.com) facilitating diverse visualization and analysis methods without requiring computational skills. In the present joint analysis, we replicated the known expansion of B lineage and the recently described expansion of natural killer (NK) cells and some cytotoxic T cells and decrease of monocytes in the CSF in MS. The previous observation of the abundance of Th1-like Th17 effector memory cells in the CSF was not replicated. Expanded CSF B lineage cells resembled class-switched plasmablasts/-cells (e.g., *SDC1*/CD138, *MZB1*) as expected. Our integrative analysis thus validates increased cell type diversity and B cell maturation in the CSF in MS and improves accessibility of available data.

**Supplementary Information:**

The online version contains supplementary material available at 10.1186/s12974-022-02667-9.

## Introduction

Multiple sclerosis (MS) is a chronic and often disabling autoimmune disease of the central nervous system (CNS) [1]. It is characterized by immune cell infiltration of the CNS resulting in local inflammation and leading to progressive loss of myelin and subsequently axons [2]. Both T and B cells contribute to MS pathogenesis [3], but the exact mechanisms of lymphocyte influx, loss of immune control and lymphocyte interaction with cells of the CNS remain unresolved [4, 5]. A common approach to studying the pathogenesis of MS has been to investigate peripheral blood mononuclear cells (PBMCs). However, the applicability of this method to study and monitor therapeutic effects of drugs targeting CNS inflammatory processes is limited because blood and its cells are distal to the CNS itself and changes in their cellular composition may not reflect pathogenic events and cellular interactions within the CNS.

Cerebrospinal fluid (CSF) has aided the diagnosis and differentiation of CNS disorders for decades but its true potential in MS remains insufficiently exploited [6]. Healthy CSF represents an ultrafiltrate of the serum and contains locally released solutes and uniquely composed leukocytes [7]. In clinical practice, this biospecimen is routinely examined for cell number and protein content. In the diagnosis of MS, it gained additional importance with the 2017 revision of the McDonald criteria which consider CSF-specific IgG oligoclonal bands as evidence of dissemination in time [8]. This illustrates the value of deep CSF-analysis for diagnosing MS and the need for an understanding of local immunological interactions. Unbiased discovery of cell types residing within the CSF including low-frequency cell populations as well as their gene expression profiles has been facilitated by the transformative single-cell RNA-sequencing (scRNA-Seq) technologies [9]. This technique may answer: (i) which cellular and molecular factors contribute to the differential activation of immune cells in MS, and (ii) is this process a peripheral or a central one [10].

Recently, a set of scRNA-seq-based studies of CSF leukocytes from patients with relapsing–remitting (RR)MS identified a location-specific composition and transcriptome of CSF leukocytes thus emphasizing the unique immune microenvironment of the CSF. One study identified signs of local interaction between T and B cells in the CSF and an increased transcriptional diversity in the CSF compared to the blood [11]. Accordingly, a T cell population expanding in the CSF expressed a transcriptional signature related to cytotoxic and effector function [12]. The different profile of CSF- compared with blood cells was supported in another study [13]. Whereas B cells were hardly detectable in the CSF of healthy controls, they clonally expanded and somatically hypermutated in MS patients and upregulated proinflammatory pathways including nuclear factor kappa B and cholesterol biosynthesis pathways. Studies also identified that monocytes in CSF transcriptionally partially resemble parenchymal microglia; a phenotype that is likely instructed by the CSF microenvironment and not ontogenetically defined [11, 14].

Despite the undeniable potential of scRNA-seq to decipher disease-specific alterations of human CSF, sizeable cohorts of patients are difficult to recruit due to the invasiveness of lumbar punctures, cost, and because CSF sampling is not practiced routinely in all MS-treatment centers. Accordingly, existing RRMS scRNA-seq datasets struggle with low patient numbers. In this joint analysis, we addressed this limitation by integrating multiple published and partially unpublished datasets. We also provide a public browsable interface, https://CSFinMS.bxgenomics.com powered by cellxgene VIP [15, 16] with enhanced functionalities for ‘personalized’ visualization and exploration. This constitutes the first freely accessible comprehensive data visualization tool for integrated CSF scRNA-seq data from treatment naïve RRMS patients and healthy individuals. In this joint analysis, we replicated the known expansion of the B lineage and the recently described expansion of natural killer (NK) cells and some cytotoxic T cells, and decrease of monocytes in the CSF. This supports recent approaches to decipher the underlying pathophysiological mechanisms in MS.

## Results

### Integrated analysis across studies improves statistical power by extending sample size

**Fig. 1:**
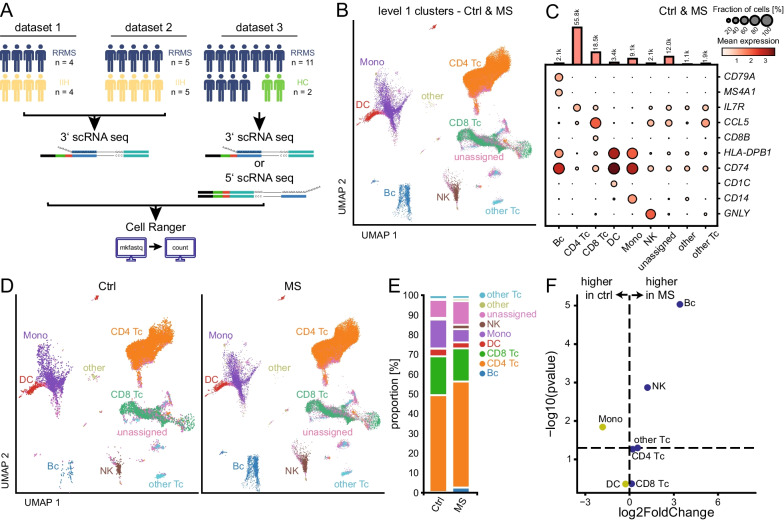
Integrated analysis facilitates characterizing the CSF immunome in treatment-naïve multiple sclerosis. **A** Schematic representation of sample cohorts, data processing, and bioinformatic analysis. Dataset 1 incorporates the data of Schafflick et al. [11], dataset 2 consists of a new muenster cohort [17] and dataset 3 incorporates the data of Ramesh et al. [13] **B** Uniform Manifold Approximation and Projection (UMAP) plot of level 1 clustering with automatic annotation based on canonical marker genes [18] (Additional file 6: Tab. S2) in the merged data set. **C** Dot plot depicting selected signature genes of level 1 clustering in merged control (Ctrl) and MS. Dot color encodes the mean expression, dot size visualizes the fraction of cells expressing the respective gene. Vertical bars on the top show the number of cells in the respective group. **D** Stacked bar plot showing the proportion of the level 1 cell types in Ctrl and in MS. **E** Split UMAP plots comparing level 1 clustering in Ctrl (left) and MS (right). **F** Volcano plot depicting differences of cluster abundance in MS compared to Ctrl. Clusters highlighted in blue are higher expressed in MS, yellow ones are more expressed in the Ctrl group. Fold change (log2) is plotted against t-test *p* value (-log10). The horizontal line visualizes the significance threshold of *p* = 0.05. Cluster key: Mono, monocyte cluster; DC, dendritic cell cluster; Bc, B cell cluster; CD4 Tc, CD4^+^ T cell cluster; CD8 Tc, CD8^+^ T cell cluster; other Tc, other T cell cluster. Un_assigned and other, cells lacking definitive assignment

The aim of this study was to substantiate previous scRNA-seq results from CSF cells by integrating existing datasets from RRMS patients and controls across studies. We first obtained and analytically integrated two datasets including 4 RRMS vs 4 control patients (dataset 1) [11], and 11 RRMS vs 2 control patients (dataset 3) [13]. In addition, we incorporated 5 treatment-naïve RRMS vs 5 control patients (dataset 2, partially published in [17]) thus achieving a total of 20 RRMS and 11 control patients (Fig. 1A).

Inclusion criteria were largely comparable between studies which enrolled both CIS and RRMS patients in relapse and diagnosed according to the 2017 McDonald criteria (Methods). None of the patients had received immunomodulatory treatment. The diagnosis was either confirmed by follow-up within 4 weeks [11] or definitively diagnosed at study entry [13]. The study by Schafflick et al. [11] explicitly excluded concomitant autoimmune diseases. Control patients notably differed between studies and encompassed 9 patients with idiopathic intracranial hypertension (IIH; dataset 1 + 2) and 2 healthy controls (dataset 3) (Additional file 5: Tab. S1). We collected available clinical meta-data from all studies and found no significant differences in the distribution of age and sex across the cohorts (Additional file 5: Tab. S1, Additional file 1: Fig. S1A). Specifically, the median age of the MS patients in cohort one was 38 years, in the second cohort was 44 years and in the third cohort was 44 years (p = 0.66). The median age of the IIH patients in cohort one was 28.5 years, in cohort two 35 years and the median age of the healthy controls in cohort three was 30 years (*p* = 0.82). We then analytically integrated all available single cell data resulting in 80,187 RRMS-derived single-cell transcriptomes (subsequently denoted as ‘cells’ for simplicity) and 25,764 control cells (Additional file 1: Fig. S1B; Additional file 5: Tab. S1). Data were processed with the Cell Ranger / Seurat v4.0 bioinformatics pipeline [18]. The scRNA-seq chemistry for the first and second cohort was 10 × 3' and for the third cohort was mixed between 10 × 3′ and 10 × 5′ (Additional file 5: Tab. S1). We first tested for gross systematic technical bias between the datasets. The median cell number per sample in cohort one was 2817 (IQR_25–75_ = 1338–3739), in the second cohort was 3,555 (IQR_25–75_ = 1553–5096) and in the third cohort was 3269 (IQR_25–75_ = 2364–4844) (Additional file 1: Fig. S1C). Mean genes detected per cell in cohort one was 1005, in the second cohort was 853 and in the third cohort was 1077 (Additional file 5: Tab. S1, Additional file 1: Fig. S1C). Batch effects were removed using SCTranformation (Additional file 2: Fig S2).

We then performed a principal component analysis (PCA) using *all* genes detected in *all* cells across *all* samples (‘pseudo-bulk’). We found that the separation between samples did not systematically differ across different cohorts although there were two individual outliers (Additional file 3: Fig. S3). These two outliers from dataset 2 were not characterized by apparent differences in clinical terms and were therefore included in further analyses. This argues against major systematic technical bias across cohorts and scRNA-seq chemistries.

### Accessible bioinformatic tool allows querying cellular and transcriptional patterns in the CSF in MS

Following this basic technical and clinical validation, we generated a freely accessible visualization and analysis tool to facilitate investigating CSF cells in MS vs control without prior bioinformatic expertise (http://CSFinMS.bxgenomics.com/). Specifically, this tool provides two levels of cell type annotation based on Multimodal Reference Mapping (Seurat v4) inferred from a CITE-seq reference dataset (162,000 blood cells, 228 antibodies) [18]: (i) Level 1 annotation of 9 general cell types and (ii) Level 2 annotation with 31 individual subclusters and thus more detailed annotation of cell subsets. On each level, marker genes and genes differentially expressed between RRMS vs controls can be interactively queried and visualized (Methods).

The combined dataset including 105,951 CSF single-cell transcriptomes was projected onto a latent space and visualized using Uniform Manifold Approximation and Projection (UMAP) plots defined by the reference dataset [18]. Using the first annotation level (Fig. 1B, Additional file 6: Tab. S2), we identified nine main clusters (named level 1 clusters) annotated as B cells (Bc; *n* = 2125), CD4 T cells (CD4 Tc; *n* = 55,814), CD8 T cells (CD8 Tc; *n* = 18,488), dendritic cells (DC; *n* = 3440), monocytes (Mono, *n* = 9082), natural killer cells (NK; *n* = 2059) and other T cells (*n* = 1909). Cells lacking definitive assignment (other *n* = 1074, unassigned *n* = 11,960) were not further considered in the analysis. Annotation was congruent with the expression of canonical cell type marker genes (Fig. 1C) in these level 1 clusters (Bc: *CD79A, MS4A1*; CD4 Tc: *IL7R*; CD8 Tc: *CCL5, CD8B*; DC: *HLA-DBP1, CD74, CD1C*; Mono: *HLA-DBP1, CD74, CD14*; NK: *GNLY*) supporting the adequacy of our annotation. In accordance with previous studies of the CSF [10, 12, 14], all detected cells in this combined CSF dataset were of hematopoietic origin and T cells were the most abundant cell type with a preponderance of CD4 T cells outnumbering myeloid and B lineage cells (Fig. 1D).

Next, we aimed to identify gross MS-associated changes and therefore tested for differential level 1 cluster abundance between RRMS patients and controls (Additional file 7: Tab. S3). Already in a simple qualitative comparison, the frequency of B cells was higher in MS vs control samples (Fig. 1D) as described previously [11, 13]. The proportion of B cells in the integrated data set was 3% in RRMS patients vs 0% in healthy controls (Fig. 1E). Quantification also identified a general myeloid-to-lymphoid shift with proportions of B cells, T cells and NK cells significantly expanded at the expense of the Mono cluster (Fig. 1E, F). These proportional alterations in MS-derived CSF cells had also been described previously [11, 13].

### Integrating datasets allows characterizing rare CSF cell populations through higher resolution clustering

**Fig. 2:**
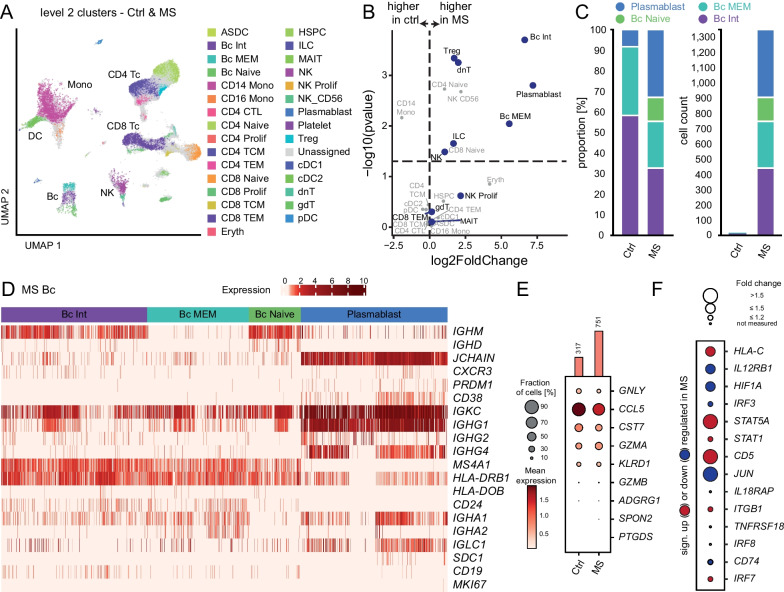
Higher resolution characterization of CSF cells in treatment-naïve MS vs control patients. **A** UMAP plot of level 2 clustering in merged control (Ctrl) and MS with automatic annotation based on canonical marker genes [18] (Additional file 8: Tab. S4). Cluster key (as not listed Fig. 1): ASDC, AXL^+^ SIGLEC6^+^ dendritic cells; CD4 CTL, CD4^+^ cytotoxic T lymphocyte; CD4/CD8 TCM, CD4^+^/CD8^+^ memory T cell; CD4/CD8 TEM, CD4^+^/CD8^+^ T effector memory cell; Eryth, erythrocytes; HSPC, hematopoietic stem and progenitor cell; ILC, innate lymphoid cell; MAIT, mucosal associated invariant T cell; NK, natural killer cell; Treg, regulatory T cell; cDC1/DC2, conventional dendritic cell; dnT, double-negative T cell; gdT, gamma delta T cell; pDC, plasmacytoid dendritic cell. **B** Volcano plot visualizing cluster abundance in Ctrl compared to MS of level 2 clustering plotted as fold change (log2) against t-test *p* value (-log10). Please note that the number of cells in some Ctrl clusters (e.g., Bc naïve) was insufficient to calculate the fold change. Clusters highlighted in blue are upregulated in MS and represent clusters of interest. **C** Left stacked bar plot showing the relative distribution of B cell subclusters in Ctrl compared to MS. Each cluster is set to 100% irrespective of its abundance. Right stacked bar plot showing the total number of B cell clusters in Ctrl vs MS. **D** Heatmap visualizing expression of immunoglobulin and other selected genes in B cell clusters. Expression values were normalized per gene with 0 reflecting the lowest and 10 reflecting the highest expression, visualized via color intensity. **E** Dot plot of selected signature genes for gdT cells in MS compared to Ctrl. Dot size represents the fraction of cells expressing the gene, color intensity shows the mean expression. **F** Precomputed differential expressed genes by cellxgene VIP of selected marker genes of gdT cells. Color coding shows significant up- (red) or downregulation (blue) in MS compared to Ctrl, dot size encodes the fold change. FDR > 0.1

We next asked whether higher resolution clustering — facilitated by increased cell numbers after integration — would reveal previously unrecognized MS-associated changes. We therefore clustered and annotated all 105,951 CSF single-cell transcriptomes using deeper level 2 clustering (Fig. 2A) and annotation from a reference dataset [18]. This considerably increased cellular granularity to 31 clusters (Fig. 2A, Additional file 8: Tab. S4). For example, we identified 4 level 2 subsets of cells ascribed to the B cell lineage: B memory (Bc MEM), B intermediate (Bc Int), B naïve cells (Bc Naïve), and antibody secreting cells; referred to as plasmacells/-blasts (plasma) for simplicity. Myeloid lineage cells separated into 5 subclusters (CD14 Mono, CD16 Mono, cDC2 (myeloid/conventional DC1), ASDC (AXL^+^ SIGLEC6^+^ DCs), cDC1) based on the expression of subset marker genes (Fig. 2A, Additional file 8: Tab. S4). CD4 T cells separated into 6 subclusters (CD4 Naive, Treg, CD4 TCM (central memory), CD4 TEM (effector memory), CD4 CTL (T cells with cytotoxic activity), CD4 Prolif (proliferating)) and CD8 T cells into 4 individual clusters (CD8 Naive, CD8 TCM, CD8 TEM, CD8 Prolif). There was a small subcluster of double-negative T cells (dnT) in the CD8-cluster. Several smaller clusters separated from the larger clusters (HSPC, MAIT, platelet). Notably, smaller clusters of such identity had not previously been detected in CSF datasets [11, 13]. We thus replicated cell types known from previous CSF scRNA-seq studies, but also demonstrated the potential of joint analyses to identify rare CSF cell populations.

### Joint analysis of single CSF cell transcriptomes helps confirming and refuting previous findings in CSF in MS

We next quantified disease-associated compositional and transcriptional changes at level 2 clustering (Fig. 2B, Additional file 9: Tab. S5). Again, the increase of B lineage clusters annotated as memory and / or class-switched was the most pronounced alteration among MS-derived cells (Fig. 2B) but the low number of these clusters in control patients obviated calculating differential gene expression. The plasma cluster and other B cell clusters were almost exclusively detected in the CSF of MS patients (Fig. 2C) (plasma: *n* = 443 in MS vs *n* = 0 in control). These changes had been described previously [11, 19] and were thus replicated at higher resolution in the present integrated dataset.

B lineage cells are nearly absent from non-diseased CSF [10]. In our joint analysis including 11 control patients, we annotated 12 total cells as B cells in the combined control dataset compared to 1346 B cells among MS CSF cells. Aiming to better understand B cells in the CSF in MS, we next plotted B cell-associated gene sets across the respective clusters. In accordance with our annotation, naïve B cells expressed *IGHM* and *IGHD* while the plasma cluster expressed *JCHAIN*, *CXCR3*, *PRDM1*, *CD38,* and class-switched immunoglobulin chain genes (e.g., *IGHG*), and lacked *MS4A1* (Fig. 2D). Antigen presentation-associated genes (e.g., *HLA-DRB1, HLA-DOB*) and *MS4A1*/CD20 were expressed across all non-plasma B cell clusters and upregulation of *CD24* and downregulation of *IGHM* characterized memory B cells (Fig. 2D). Notably *IGHA* gene expressing plasma cells recently described in MS [20] were also detected. When performing a detailed transcriptional characterization of B cells clusters, we observed a phenotype indicative of antibody secreting cells (Fig. 2D). Our integrated transcriptional characterization thus identifies B cells across a developmental continuum specifically in the CSF in MS.

We next focused on compositional changes described previously, but not replicated in our integrated dataset; this was true for γδ T cells (gdT cells). The merged dataset showed a non-significant increase in gdT cells in the CSF of MS patients (751 cells in MS vs 317 cells in control patients (Fig. 2B)) while a reduction had been described previously [13]. Differences in cell annotation may account for some of the differences. *GNLY, CCL5* and *CST7* were all downregulated in the gdT cells of the integrated MS data (Fig. 2E) indicating a potential reduction in cytotoxic potential. Upregulation of *HLA-C* in this cluster in MS was in line with previous results [13], while genes *IL12RB1*, *HIF1A,* and *IRF3* were only downregulated and *STAT5A*, *STAT1,* and *CD5* were only upregulated in the integrated data (Fig. 2F). Our joint analysis thus enables a deeper transcriptional characterization with increased confidence and replicates some, but not all previously described changes.

### Deep characterization of rare cell types facilitated by integrated CSF scRNA-seq data analysis

We next aimed to better characterize the cell types with profound differential abundance in MS vs control comparisons with specific focus on MS-related changes not previously reported in single cell studies.

**Fig. 3:**
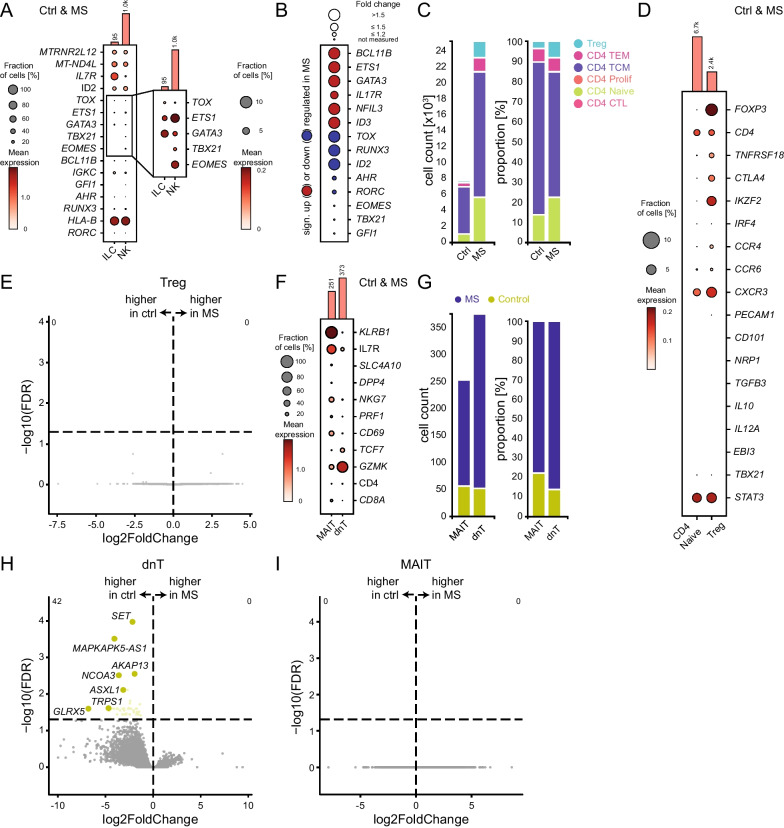
Characterizing rare cell populations in the integrated CSF cell dataset in MS. **A** Dot plot of selected genes for smaller subclusters of innate lymphoid cell (ILC) and natural killer cell (NK) in merged control (Ctrl) and MS. FDR > 0.1. To better visualize the differences, the right dot plot shows a zoom-in of selected genes with an adjusted scale. **B** Precomputed differential expressed genes by cellxgene VIP of selected genes of ILCs in MS compared to Ctrl. Color coding shows significant up- (red) or downregulation (blue), dot size encodes the fold change. FDR > 0.1. **C** Left stacked bar plot showing the total number of depicted T cell clusters in Ctrl vs MS. Right stacked bar plot shows the relative distribution of T cell subclusters in Ctrl compared to MS. Each cluster is set to 100% irrespective of its abundance. **D** Dot plot of selected genes characterizing the Treg cluster compared to CD4 naïve cluster in merged Ctrl and MS. FDR > 0.1. **E** Volcano plot of precomputed differential expressed genes by cellxgene VIP of Treg cells in MS compared to Ctrl. The horizontal line depicts the significance threshold (FDR, false discovery rate = 0.05). **F** Dot plot of selected signature genes of mucosal associated invariant T cells (MAIT) and double-negative T cells (dnT) in merged Ctrl and MS. **G** Stacked bar plot showing the relative distribution of MAIT cells and dnT cells in MS compared to Ctrl. **H** Volcano plot of precomputed differential expressed genes by cellxgene VIP of dnT cells in MS compared to Ctrl. Please note that yellow highlighted genes are higher expressed in the Ctrl and are only applied to significantly upregulated genes. The horizontal line depicts the significance threshold (FDR, false discovery rate = 0.05). **I** Volcano plot of differential expressed genes in MAIT cells in MS compared to Ctrl; no gene identified. FDR > 0.1

Specifically, an expansion of cell clusters annotated as innate lymphoid cells (ILC) and double-negative (dnT; i.e., CD4^−^CD8^−^) T cells was newly identified (Fig. 2B). The ILC cluster was more abundant in MS patients than in controls (Ctrl *n* = 13 vs MS *n* = 82 cells) and expressed *MTRNR2L12* and *MT-ND4L* (Figs. 2B, 3A). ILCs differ from NK cells in their transcriptional regulation by *IL7R, ID2, TOX, ETS1,* and *GATA3*, whereas NK cells depend on the transcription factors TBX21 and EOMES [21] (Fig. 3A). *BCL11B, ETS1, GATA3, IL17R, NFIL3,* and *ID3* were upregulated in ILCs of MS patients, whereas *TOX, RUNX3, ID2,* and *AHR* were downregulated (Fig. 3B) potentially indicating loss of regulatory mechanisms.

Differential abundance of Tregs had previously been only marginally significant [11]. The merged dataset showed that the Treg cluster was more abundant in MS patients (cell count Ctrl *n* = 288 vs MS *n* = 2086) (Fig. 3C) and was characterized by *FOXP3*, *CD4*, *TNFRSF18,* and *CTLA4* [22] (Fig. 3D). Expression of *IKZF2*, *IRF4*, *CCR4*, *CCR6,* and *CXCR3* was indicative of induced Tregs (iTreg) [23], and further confirmed by the absence of the natural Treg (nTreg) markers *PECAM1*, *CD101,* and *NRP1*. nTregs are unstable and transit into a Th17-like phenotype under inflammatory conditions and the presence of IL-6 [24]. In contrast, iTregs retain at least temporarily their immunoregulatory capacity despite an autoreactive environment [25]. In our integrative dataset, Treg cells did not express genes of anti-inflammatory cytokines (e.g., *TGFB3*, *IL10*, *IL12A*, *EBI3*) or the transcription factor for the Th1-lineage T-bet (*TBX21*), but exposed the transcription factor for Th17 lineage *STAT3* (Fig. 3D), indicating a Th17-like rather than Th1-like phenotype [26–28]. Despite the higher abundance of Treg cells in the CSF of MS patients, none of the genes were significantly differentially expressed (Fig. 3E). Considering the potential of iTreg to suppress ongoing autoimmune response [29], Treg expansion may reflect local regulatory mechanisms in the CSF in MS. These mechanisms are likely exhausted by persistent autoreactive mechanisms and thus depicts a potential therapeutic approach.

Next, we characterized the clusters annotated as mucosal associated invariant T cells (MAIT) and dnT (Fig. 3F). MAIT expressed *KLRB1*, *IL7R*, *SLC4A10,* and *DPP4* and dnT lacked expression of CD4 and CD8. We detected 373 dnT cells which were preferentially MS-derived (Ctrl *n* = 52 vs MS *n* = 321 dnT cells) (Fig. 3G). Despite the higher frequency of dnT cells in MS, no gene was upregulated while 42 genes were downregulated (e.g., *SET*, *MAPKAPK5-AS1*, *NCOA3*, *AKAP13*, *ASXL1*, *TRPS1*, *GLRX5*) (Fig. 3H). The MAIT cluster consisted of 251 cells, again with preferential detection in the MS CSF (Ctrl *n* = 56 vs MS *n* = 195 MAIT cells) (Fig. 3D). No genes were significantly differentially expressed (Fig. 3I). Overall, this set of differentially abundant clusters can be summarized as cells with both innate and cytotoxic phenotypes but also regulatory function expanding in the CSF in MS across studies. An expansion of cytotoxic phenotype CD4 T cells (albeit not ILC, MAIT, dnTc) had been described previously [11] and this may reflect similar changes annotated differently.

### Integrated analysis reveals inflammatory phenotype of CD16^+^ monocytes

**Fig. 4:**
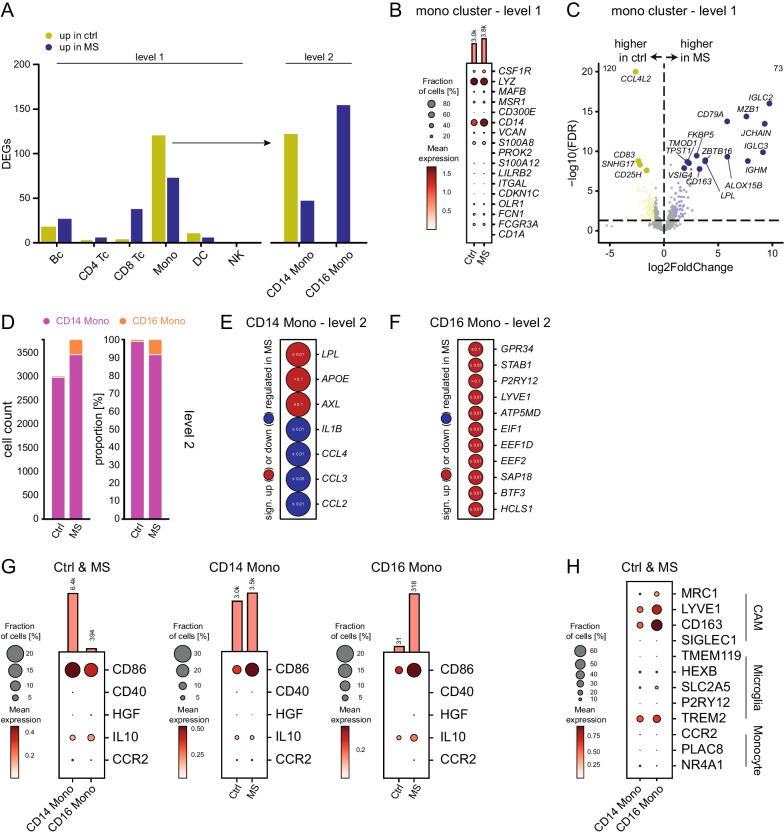
Deeply characterizing monocyte cells in the CSF of MS and control patients. **A** Grouped bar plot of the absolute count of differential expressed genes (DEGs) in listed cell types in level 1 and level 2 clustering. **B** Dot plot of selected genes for the total Mono cluster (level 1) in MS compared to control (Ctrl). **C** Volcano plot showing differential expressed genes of the Mono cluster (level 1) in MS compared to Ctrl plotted as fold change (log2) against *p* value (-log10, FDR). Please note that yellow highlighted genes are higher expressed in the Ctrl and blue ones are more expressed in MS. The upregulation of B cell typical genes we interpret as an artifact here due to miss annotation. **D**  Left stacked bar plot showing the cell count of CD14^+^ and CD16﻿^+^ monocytes in MS compared to Ctrl. Right s-tacked bar plot showing the proportion of CD14^+^ and CD16^+^ monocyte cells in MS compared to Ctrl. Both monocyte cell clusters are set to 100% irrespective of their total abundance, thereby not allowing a quantitative comparison. **E** Precomputed differential expressed genes by cellxgene VIP of selected genes with a focus on phagocytosis, lipid metabolism and trafficking-associated genes in CD14^+^ and **F** CD16^+^ monocyte cells. Color coding shows significant up- (red) or downregulation (blue), dot size encodes the fold change. Small white numbers represent the FDR. Fold change > 1.5. **G** Dot plot of gene expression of activation markers and anti-inflammatory cytokines in CD14^+^ and CD16^+^ monocytes in MS and Ctrl. **H** Dot plot depicting expression of signature genes for CNS-associated macrophages (CAM) [40, 41], microglia [53] and monocyte-derived cells [40] in CD14^+^ and CD16^+^ monocytes in Ctrl and MS

Overall, clusters annotated as monocytic cells (level 1) showed the greatest number of differentially expressed genes in MS vs control comparison (Fig. 4A) across several studies [11, 13] indicating preferential phenotypic alterations in myeloid lineage cells in the CSF in MS. In the level 2 clustering, this was especially pronounced in CD16^+^ monocytes, which upregulated 156 genes in MS and were relatively more abundant in MS (Fig. 4A). We therefore next aimed to capitalize on the potential of our integrated dataset to better understand how MS affects CSF cell types of such phenotype.

We first characterized the total Mono cluster (level 1) classified by genes like *CSF1R*, *LYZ*, *MAFB*, M*SR1, and CD300E* (Fig. 4B) [30]. Genes associated with lipid metabolism like *ALOX15B* and *LPL* or involved in immunoregulation like *FKBP5* and *CD163* were among the 73 upregulated genes in the overall Mono cluster of MS patients (Fig. 4C). Genes like *IGLC2*, *MZB1*, *JCHAIN,* and *CD79A*, which are known B cell markers, were upregulated in the Mono cluster which could represent remnant mis-annotated B cells in the Mono cluster (Fig. 4C).

Next, we analyzed the subclusters of the CD14 and CD16 monocytes (level 2). In the past, monocytes and their activation markers were mainly studied in PBMC [31–33]. CD16^+^ and CD14^+^ monocytes — especially in the CSF of MS patients — are not yet well characterized.

In our joint analysis of RRMS and IIH/control CSF of three independent datasets, the number of CD14^+^ monocytes was higher than the number of CD16^+^ monocytes in both control and MS (Fig. 4D). While CD14^+^ monocytes are known to decrease in the blood of MS patients [34], the total number of CD14^+^ monocytes was increased in the CSF of MS patients compared to controls supporting the independence of blood and CSF compartments. The CD14^+^ monocytes in the MS samples were characterized by an upregulation of genes associated with phagocytosis and lipid metabolism like *LPL*, *APOE,* and *AXL* [35]*,* an observation reminiscent of the phenotypes displayed by microglia at the rims of the chronic active lesions [36, 37] (Fig. 4E). Furthermore, we found a downregulation of trafficking-associated transcripts in the CD14 Mono cluster (*IL1B, CCL4, CCL3, CCL2)* (Fig. 4E). We interpret this downregulation as supportive of the classification of CD14^+^ monocytes as rather “CSF-derived” as opposed to “periphery-derived” as described by Schafflick et al. [11].

CD16^+^ monocytes in CSF were more abundant in MS than in the controls (Fig. 4D). *GPR34*, *STAB1*, *P2RY12,* and *LYVE1* previously described in so-called border-associated macrophages (BAMs) [38], were significantly upregulated in CD16^+^ monocytes in MS. Furthermore, the CD16 Mono cluster upregulated mitochondrial (e.g., *ATP5MD*) and cell cycle-associated transcripts (*EIF1*, *EEF1D*, *EEF2*, *SAP18*, *BTF3*, *HCLS1*) indicative of proliferation (Fig. 4F).

Comparing CD14^+^ and CD16^+^ monocytes in detail, both CD14^+^ and CD16^+^ monocytes expressed activation markers (CD86, CD40) and anti-inflammatory cytokines (IL10) which were upregulated in the CSF of MS patients (Fig. 4G). Additionally, CD16^+^ and CD14^+^ monocytes showed a CNS-associated macrophage phenotype (*Mrc1, Lyve1, CD163, Siglec1*) [39–41], with CD16^+^ monocytes appearing to be more differentiated and specialized than CD14^+^ monocytes (Fig. 4H). *CCR2*, a chemokine receptor critical for crossing the blood–brain barrier [42], was significantly upregulated in the CD16^+^ monocytes but not in the CD14^+^ monocytes in MS (Fig. 4G), and may thus locally drive inflammation in MS. Similar to previous findings in the periphery of MS patients [31–33], our study identified CD16^+^ monocytes as active inducers of inflammation and considered them as one of the first cells crossing the blood–brain barrier during MS [31].

After the in-depth characterization of the individual clusters on level 1 and level 2, we were interested in further speculating on the identity of the 11,960 cells that we had defined as unassigned cells in the level 1 clustering. Based on the marker genes from level 2 clustering (Figs. 2E, 3A,D,F, 4B), the unassigned level 1 cluster showed mainly characteristics of gdT cells (*CCL5, CST7, GZMA, CD74*), ILC (*MTRNR2L12, MT-CD4L, ID2, ETS1, BCL11B, IGKC*), MAIT (*KLRB1, NKG7*), and dnT (*GZMK*) cells and less of naive CD4, Tre,g and Mono (Additional file 4: Fig. S4). This underscores that lower resolution scRNA-seq are unlikely to capture these smaller cell populations and highlights the importance of our joint analysis.

Overall, an integrated data analysis facilitates deeply characterizing disease-associated transcriptional changes in both rare and abundant cell types for a better understanding of mechanisms of MS.

## Discussion

In the present study by integrating multiple single cell datasets from the CSF in multiple sclerosis (MS), we (i) improve accessibility, and (ii) increase statistical power thereby facilitating novel findings.

Our public and browsable interface (https://CSFinMS.bxgenomics.com) constitutes the first freely accessible comprehensive data visualization tool for understanding CSF cells in MS without the need for computational skills. Users can answer biological questions, such as gene expression differences in disease, gene expression patterns among cell types, and pathway enrichment analysis of genes of interest from the above to identify the underlying mechanisms of diseases. A step-by-step guide is provided as an online documentation (interactivereport.github.io/cellxgene_VIP/tutorial/docs/index.html). The majority of figures in the manuscript are created by using the tool without assistance.

The integrated analysis replicated many features described previously, such as the expansion of B cells in the CSF of MS patients. Pronounced B cell accumulation in the CSF distinguishes MS from other inflammatory CNS diseases and differential diagnoses [19, 43, 44]. The dataset can be used to study treatment targets. Anti-CD20 antibodies are effective in MS [45] and CD20 is expressed in the ‘mid’ B cell lineage and absent from B cell progenitors and plasmablasts/-cells [46]. B lineage cells accumulating in the CSF in MS are preferentially late-stage plasmablasts/-cells. Accordingly, the expression of *MS4A1* (encoding CD20) was lower in the CSF of MS patients indicating that the antigen-experienced clones found in the CSF are not the primary target of B cell-depleting therapies [46].

The integrated analysis restricted the previously reported lower abundance of all monocytes in the CSF in MS [11, 13, 17] to a cluster annotated as classical CD14^+^ monocytes. Non-classical monocytes (CD14^+^CD16^+^) have been associated with various autoimmune diseases and contribute to blood–brain barrier (BBB) breakdown [47]. Schafflick et al. [11] classified CD16^+^ monocytes as mostly blood-derived “Mono1” and CD14^+^ monocytes as mostly CSF-derived “Mono2”. Deeper gene analysis of the latter showed similarities with homeostatic microglia [48] — a phenotype likely instructed by the CSF microenvironment and not indicating ontogenetically shared origins [49].

Considering these two examples of the integrative data set’s advantages, the aim of this study is sufficient met: it supports the previous findings and substantiates them with a higher statistical power. Nevertheless, it will be exceptionally interesting to extend our single cell study design to MS patients in remission to compare CSF single cell profile between relapse and remission. In summary, our study supplies a public and browsable tool to analyze CSF samples of MS patients and provides an essential reference point for future studies.

## Material and methods

### Study design

The current database includes a cohort of 4 MS patients and 4 control patients (dataset 1) from our published study [11], a new cohort consisting of 3 MS patients and 5 control patients published in [17] plus 2 newly recruited MS patients (dataset 2), and a cohort of 11 MS patients and 2 control patients (dataset 3) [13]. For a more detailed description of clinical and experimental procedures, we refer to the respective sections of the original articles and to Additional file 5: Tab. S1 [11, 13, 17].

### Data acquisition

Collectively, 20 relapsing–remitting multiple sclerosis (RRMS) patients and 11 controls. The latter were either healthy controls or patients with an idiopathic intracranial hypertension (IIH), a non-inflammatory condition characterized by an elevated intracranial pressure with normal CSF composition [50]. All MS patients were treatment-naïve, with RRMS in active relapse leading to the first diagnosis. The inclusion and exclusion criteria have been described previously [11, 13, 17]. Briefly, RRMS patients were diagnosed based on the revised McDonald criteria [8], and were in an active relapse and did not receive immunomodulatory or immunosuppressive disease-modifying therapy at the time of sample collection. Exclusion criteria were concurrent immunological comorbidities, particular immunological conditions (e.g., pregnancy or breastfeeding, younger than 18 years old), severe concomitant infectious diseases, or artificial blood contamination in the CSF (> 200 RBCs/µL) [11]. All participants gave their informed consent, and the studies were approved by the respective ethical committees. All available CSF single cell RNA-sequencing (scRNA-seq) data from MS and control of dataset 1, 2, and 3 were used. The exclusion criteria for excluding individual cells (not patients) from analysis were: nFeature_RNA > 200 & nFeature_RNA < 2500 & percent.mito < 0.1.

### Diagnostic puncture and cell preparation

Precise techniques and operating procedures varied in each study and can be found in detail in the respective publications. Briefly, CSF and blood were obtained within the scope of diagnostic procedures under sterile conditions and in accordance with local standard operating procedures. The additional CSF volume was centrifuged immediately after collection at 300–400×*g* for 10–15 min. The supernatant was discarded and the pellet was resuspended in an appropriate medium. The cell count was determined by a hemocytometer or manually in a Fuchs-Rosenthal chamber to achieve a proper cell concentration for scRNA-seq. The cells were stored at 4°C until processing. If processed immediately, cells were resuspended in ~ 80 µL of residual supernatant [13].

### Single-cell RNA-sequencing and library generation

Briefly, after appropriate preparation of the samples and generation of a high-quality single cell suspension, the 10 × library was constructed using reagent kits and Chromium Controller of 10X Genomics (Chromium Single Cell 3′ Library & Gel Bead Kit; 10X Genomics). The cells are partitioned by a special oil whereby single Gel Beads-in-emulsion (GEMs) are generated on a Chromium Chip. Following, the GEMs are chemically dissolved and the entailed mRNA is amalgamated with a master mix containing reverse transcription reagents. Thereby 10 × barcoded cDNA is generated and by subsequent PCR amplification a Chromium Single Cell 3′ Gene Expression library containing two special primers at each end, called Illumina paired-end constructs, is synthesized. The commonly shared 10 × barcode is followed by a Unique Molecular Identifier (UMI), which allows to define genes based on UMI counts. The resulting 10 × Barcoded libraries are compatible with standard NGS short-read sequencing on Illumina sequencers (for the respective sequencing models we refer to the respective publications). For dataset 3 from Ramesh et al. [13] sequencing libraries were prepared using 3′ or 5′ library preparation kits (10 × Genomics) (Additional file 5: Tab. S1).

### Preprocessing of sequencing data

Dataset 3 from Ramesh et al. [13] was downloaded from Gene Expression Omnibus (GEO) repository under BioProject PRJNA549712 (GEO accession no. GSE133028). Dataset 1 from our published study [11] was available from GEO repository with the accession no. GSE138266. The new cohort consisting of 3 MS patients and 5 control patients published in [17] plus 2 newly recruited MS patients (dataset 2) is available from GEO repository with the accession no. GSE163005. All the raw sequencing data were processed with the Cell Ranger pipeline v3.0.2 (10X Genomics). Raw base call (BCL) files derived by the Illumina sequencers were demultiplexed using Cell Ranger mkfastq into FASTQ files. Subsequent read alignments and transcript counting were done individually for each sample using Cell Ranger count with standard parameters. Pre-built Cell Ranger Human reference, GRCh38 (GENCODE v32/Ensembl 98) version 2020-A (July, 2020) was used for gene mapping. Raw count data were analyzed using Seurat (v4.0) bioinformatics pipeline [18]. For quality control, only cells containing transcripts for more than 200 genes and less than 2500 genes were included. Cells were omitted if they expressed > 10% mitochondrial genes. (nFeature_RNA > 200 & nFeature_RNA < 2500 & percent.mito < 0.1.)

### Batch effect removal

Gene counts were normalized using the SCTranform based normalization from Seurat (v4.0) to remove batch effect. To visualize the batch effect removal results, we first colored the UMAP plot with batch information and calculated the percentage of cells from each batch for each cell type. Then, we used regular LogNormalize method to normalize the count data and compared the results with previously SCTranformation normalized data. LogNormalize is a global-scaling normalization method that normalizes the count data for each cell by the total expression, multiplies this by a scale factor 10,000 and log-transforms the result. At last, we performed a principal component analysis (PCA) using all genes detected in all cells per sample (‘pseudo-bulk’) to create the PCA space. For each sample, we averaged the expression of each gene in all cells to create a pseudo-bulk gene expression matrix.

### Cell type prediction

To predict the cell types in our query dataset, we transferred cell type labels from the reference dataset [18] to our query dataset by following Seurat v4 Multimodal Reference Mapping pipeline (https://satijalab.org/seurat/articles/multimodal_reference_mapping.html), based on a CITE-seq reference dataset of 162,000 PBMC measured with 228 antibodies [18]. After prediction, each cell from our query dataset received cell type annotations at two levels of granularity (level 1, and level 2). Each prediction was assigned a score between 0 (low confidence) and 1 (high confidence). For the following analysis, we only used cell type prediction with high confidence (prediction score > 0.6) [51]. Then, the combined dataset was projected into a UMAP visualization defined by the reference dataset.

### Differential cluster abundance analysis

We used Student’s t-test to test for significance of differences in the proportion for each cell cluster between MS and control patients. *P* value and log2-fold change were provided in Additional file 7: Tab. S3 and Additional file 9: Tab. S5. We used R packages *ggplot2* and *ggrepel* to generate volcano plot to visualize the *p* value (*t*-test) and -log2-fold change for the difference in proportion for each cell type between MS and control patients.

### Differential gene expression analysis

For single cell-level differential expression (DE) analysis, we used NEBULA [52]. We used this approach to identify differentially expressed genes in each cluster type vs the remaining cell clusters (i.e., marker genes) and in MS vs control cells in each cell cluster (DE genes). The counts data were imported into R and two rounds of QC filtering were applied. In the first round, filtering was applied to the entire count matrix. We required: (1) a library size between 200 and 20 M, (2) genes must be expressed in at least 3 cells, and (3) cells must have at least 250 genes expressed. Additionally, mitochondrial, and ribosomal genes (all gene names containing: RP, RG, MT) were filtered out at this stage. During a second round of filtering, we required a minimum of 10% of all the cells to express a gene, a minimum of 3 cells per subject, and a minimum of 2 subjects per group. Any gene where both groups have a 90th percentile of expression at 0 was filtered out. Additionally, batch effect, age, sex and percentage of mitochondrial genes were considered as covariates/confounding factors and adjusted in the analysis.

### Data visualization

We deposited the combined dataset into single-cell RNA-seq visualization tool cellxgene VIP [15, 16] to share the data and allow readers to further explore and visualize it. The tool is available at https://CSFinMS.bxgenomics.com. The online tutorial, https://bit.ly/3z4jRo8 provides the general guidance on using the tool. In addition, the following will outline few use cases to answer particular biological questions. The following abbreviations are used throughout the cellxgene VIP tool: MS, Multiple Sclerosis; CSF, Cerebrospinal Fluid; DEG, differentially expressed genes; Lev1, level 1; Lev2, level 2; F, female; M, male; VIP, Visualization In Plugin.

## Supplementary Information

**Additional file 1: Figure S1. **Patient/Data characteristics. (A) Age and gender composition of all control (Ctrl, n = 11) and MS patient (n = 20) is shown separately for each dataset. (B) Obtained cell count [× 10^4^] in the cohort of Ctrl and MS patients in the total sequenced dataset. (C) Whisker plots depicting the measured cell count [× 10^4^] per sample as well as the genes [× 10^2^] per cell divided by dataset.

**Additional file 2: Figure S2. **Correction for batch effect by SCTranform normalization. (A) The UMAP shows no obvious batch effect after SCTranform normalization. The stacked bar plot next to the UMAP shows the percentage of cells from each batch per cell type. (B) The UMAP depicts the batch effect after LogNormalize normalization. (B) The stacked bar plot next to the UMAP shows the percentage of cells from each batch per cluster. For example, as shown in the bar plot, Cluster 0 and 5 mainly contain cells from Dataset 3, while Cluster 2 mainly contains cells from Dataset 1 and Cluster 7 and 9 mainly contain cells from Dataset 2. Dataset 1: Schafflick et al. [11], dataset 2: new muenster cohort and Heming et al. [17], dataset 3: Ramesh et al. [13].

**Additional file 3: Figure S3. **No batch effect identified based on pseudo-bulk principal component analysis. Pseudo-bulk principal component analysis (PCA) shows no obvious batch effect across the three datasets. To generate pseudo-bulk gene expression matrix, we averaged the expression of each gene in all cells from one sample. Dataset 1: Schafflick et al. [11], dataset 2: new muenster cohort and Heming et al. [17], dataset 3: Ramesh et al. [13].

**Additional file 4: Figure S4. **Characterization of unassigned cluster level 1. Depicted is the expression level of marker genes for listed cell types in the unassigned cluster of level 1 of controls (Ctrl) and MS patients. Marker genes were selected based on level 2 cluster analysis; the corresponding Figures are linked. Dot color encodes the mean expression; dot size visualizes the fraction of cells expressing the respective gene.

**Additional file 5: Table S1. **Subject demographics and technical information on scRNA-Seq. (Table 1.1) Composition of the three datasets, subject demographics of included multiple sclerosis (MS, n = 20) and control (IIH/HC = Ctrl, n = 11) patients, MRI features and CSF findings are listed. For dataset 3, non-included samples from Ramesh et al. [13] are listed (grayed out) and their exclusion criteria are shown. See methods for the primary source of the three datasets. Column legend: IIH = idiopathic intracranial hypertension, MS = multiple sclerosis, CIS = clinically isolated syndrome, f = female, m = male, rel_gd = relapse, lesions with gadolinium enhancement detected on magnetic resonance imaging (MRI), rel_nogd = relapse, no lesion with gadolinium enhancement on MRI, MS-typical MRI = MS-typical lesion pattern in characteristic locations (ovoid periventricular lesions/Dawson fingers, cortical or juxtacortical, infratentorial and spinal cord) (1) / no Ms-typical lesion pattern (0), active = active relapse, Gd +  = gadolinium detected (1) or not detected (0) in MRI lesion, months from onset = months from first symptoms until sample collection, cell = cell count/µL obtained via lumbar puncture, lympho = lymphocytes/µL of cerebrospinal fluid (CSF), granulo = granulocytes/µL of CSF, rbc = red blood cell count/µL CSF, protein = obtained protein concentration in mg/L CSF, lactate = CSF lactate in nmol/L, glucose = CSF glucose in mg/dL, OCB +  = any oligoclonal bands (OCB) detected in CSF (1) or not detectable (0). Barrier disruption = no barrier disruption (0) or present barrier disruption (1). CSF index = derived from the Reiber scheme [54] (IgG CSF/IgG serum index compared to the albumin CSF/albumin serum index), no barrier disruption (none), intrathecal immunoglobulin synthesis (Ig only), intrathecal immunoglobulin synthesis plus barrier disruption (barrier_and_Ig). IgG-index > 0.7 = IgG synthesis in the CSF. All parameters were obtained within 24 h of CSF sampling. (Table 1.2) Technical information on scRNA-seq results of all patients (MS, n = 20) and healthy controls (Ctrl, n = 11) included in the study are listed. The sequencing technique for generation of the 10 × libraries is listed for the different datasets (10 × Genomics scRNA-seq kit version). Furthermore, the total number of measured cells after sequencing and genome alignment (total cells per sample) and the average number of detected genes per cells (average gene per cell) used for downstream analysis is depicted.

**Additional file 6: Table S2. **Marker genes / differentially expressed genes_cell type level 1. (Table 2.1) Overview of marker genes and references used to annotate and specify the respective clusters. (Table 2.2–2.10) Depicted are the differentially expressed genes in each cluster compared to the remaining clusters of the cell type level 1 (marker genes). Listed for each gene is the Log fold change (log2-fold change), the *p*-value und the adjusted p-value (false discovery rate). We used the Welch’s t-test (cellxgene or diffxpy) for the discovery of differentially expressed genes between two groups of cells. A minimum of 10 cells in each group was required for calculation.

**Additional file 7: Table S3. **DE genes / Differentially expressed genes MS vs control cell type level 1. Depicted are the differential expressed genes in MS compared to control (Ctrl) for each cluster of cell type level 1 (DE genes). Listed for each gene is the Log fold change (log2-fold change), the *p*-value und the adjusted p-value (false discovery rate). We used the Welch’s t-test (cellxgene or diffxpy) for the discovery of differentially expressed genes between two groups of cells. A minimum of 10 cells in each group was required for calculation.

**Additional file 8: Table S4. **Marker genes / Differential expressed genes_ cluster cell type level 2. (Table 4.1) Overview of marker genes and references used to annotate and specify the respective clusters. (Table 4.2–4.30) Depicted are the differential expressed genes in each cluster compared to the remaining clusters of the cell type level 2 (marker genes). Listed for each gene is the Log fold change (log2-fold change), the *p*-value und the adjusted p-value (false discovery rate). We used the Welch’s t-test (cellxgene or diffxpy) for the discovery of differentially expressed genes between two groups of cells. A minimum of 10 cells in each group was required for calculation.

**Additional file 9: Table S5. **DE genes / Differential expressed genes MS vs Control cell type level 2. Depicted are the differential expressed genes in MS compared to control (Ctrl) for each cluster of cell type level 1 (DE genes). Listed for each gene is the Log fold change (log2-fold change), the *p*-value und the adjusted p-value (false discovery rate). We used the Welch’s t-test (cellxgene or diffxpy) for the discovery of differentially expressed genes between two groups of cells. A minimum of 10 cells in each group was required for calculation.

## Data Availability

All processed, unmodified scRNA-seq data (differential expression data) are included as Additional Tables. Technical scRNA-seq information and data tables with details of the included patients are included as Additional Tables. The dataset supporting the conclusions of this article is available for download at https://doi.org/10.5281/zenodo.6910635, which can be explored in the cellxgene VIP tool, http://CSFinMS.bxgenomics.com.

## Abbreviations

ASDC: AXL^+^ SIGLEC6^+^ CD11c^−/low^ dendritic cell
Bc: B cells
CITE-seq: Cellular indexing of transcriptomes and epitopes by sequencing
TCM: Central memory T cell
CNS: Central nervous system
CSF: Cerebrospinal fluid
CIS: Clinically isolated syndrome
CD: Cluster of differentiation
Ctrl: Control
cDNA: Copy deoxyribonucleic acid
DC: Dendritic cells
DE: Differential expression
DEG: Differentially expressed genes
dnT: Double-negative T cell
TEM: Effector memory T cell
Eryth: Erythrocytes
FDR: False discovery rate
f: Female
gdT cells: Gamma delta T cells
GEM: Gel beads-in-emulsion
GEO: Gene expression omnibus
granulo: Granulocyte
HC: Healthy control
HSPC: Hematopoietic stem and progenitor cell
IIH: Idiopathic intracranial hypertension
Ig: Immunoglobulin
iTreg: Induced regulatory T cell
ILC: Innate lymphoid cell
Int: Intermediate
IQR: Interquartile range
lympho: Lymphocyte
MRI: Magnetic resonance imaging
m: Male
MEM: Memory
mRNA: Messenger ribonucleic acid
MT: Mitochondrial
Mono: Monocytes
MAIT: Mucosal associated invariant T cell
MS: Multiple sclerosis
cDC: Myeloid/conventional dendritic cell
NK: Natural killer
nTreg: Natural regulatory T cell
NEBULA: Negative binomial mixed model using a large-sample approximation
NGS: Next generation sequencing
OCB: Oligoclonal bands
PBMC: Peripheral blood mononuclear cell
plasma: Plasmacell
pDC: Plasmacytoid dendritic cell
PCA: Principal component analysis
Prolif: Proliferation
QC: Quality control
BCL: Raw base call
rbc: Red blood cell
Treg: Regulatory T cell
rel_gd: Relapse, lesions with gadolinium enhancement detected on MRI
rel_nogd: Relapse, no lesions with gadolinium enhancement on MRI
RRMS: Relapsing–remitting multiple sclerosis
RG: Ribosomal gene
RP: Ribosomal protein
scRNA-seq: Single-cell RNA-sequencing
TCL: T cell with cytotoxic activity
Th cell: T helper cell
UMAP: Uniform manifold approximation and projection
UMI: Unique molecular identifier
VIP: Visualization in plugin
